# S197. OBSTETRIC COMPLICATIONS, NEUROCOGNITION, AND SCHIZOPHRENIA

**DOI:** 10.1093/schbul/sby018.984

**Published:** 2018-04-01

**Authors:** Bjorn Rund, Charlotte Teigset, Christine Mohn

**Affiliations:** University of Oslo

## Abstract

**Background:**

Schizophrenia is a disorder with a heterogeneous genetic and neurobiological background that influences early brain development. The symptoms is the behavioural outcome of deviations in early neurodevelopment, including prenatal insults such as obstetric complications (OC). OC have been linked to an increased risk for schizophrenia in offspring, especially in early-onset schizophrenia (EOS). Extensive cognitive deficits occur in EOS, whereof executive function is one of the best documented. Cognitive dysfunction reflects underlying abnormalities in the brain neurodevelopment, and is considered to be an intermediate variable between OC and schizophrenia. Our research group (Teigset et al, 2016) is the only study that has investigated the relationship between OC and cognition in EOS. This study aimed to examine the frequency of OC in EOS compared to controls, and also investigate the relationship between OC and neurocognitive dysfunction. In the present presentation we will focus upon executive function and report the findings when comparing the same sample of patients and controls as in the Teigset et al study.

**Methods:**

Nineteen EOS patients and 53 healthy controls were tested with the MATRICS Consensus Cognitive Battery(MCCB), and two tests for assessment of executive functioning. The selected subtests for measuring executive function were the D-KEFS Color Word Interference Test (Stroop) and the Wisconsin Card Sorting Test. WCST assesses perseverative responses and failure to maintain set, and the Stroop assesses time in seconds for completing the Inhibition and Switching conditions. The cognitive measures were combined with data from the Norwegian Birth Registry (NMBR). Information on OC was collected from the NMBR containing information about all births in Norway, including information about maternal health before and during pregnancy, and any complications arising during pregnancy or birth. The registry includes information about medication during pregnancy, labor interventions, birth complications, maternal complications after birth, whether this was a live birth, any diagnoses in the child or evidence of congenital abnormalities.

**Results:**

Group differences in OC were studied with Student’s t-tests and Chi-square tests. The association between OC and cognitive function were studied using linear regression analyses. The results indicated no group differences in OC in EOS and healthy controls. However, a shorter gestational length in the EOS group led to significant decreases in the overall neurocognitive composite score (MCCB), in processing speed and in the two executive function tasks.

**Discussion:**

Our findings indicate that a shorter gestational length did not increase the risk for developing EOS, but was significantly associated with the cognitive difficulties in this group. In particular, executive functioning were affected, a finding in line with those of Brown et al (2009), showing that prenatal infections were associated with impaired executive function. Interestingly, reductions in neurocognitive performance among those exposed to OC was less extensive in the healthy control group with the same labor-conditions, which may indicate a greater effect of OC on neuropsychological development in schizophrenia. In conclusion, gestational length does not increase the risk for developing EOS, but significantly affects the cognitive difficulties - particularly executive function - seen among cases.

**References:**

1. Brown et al. (2009) Am J Psych, 166: 683–690.

2. Teigset et al. (2016). Psych Res, 244: 78–85.

